# Pharmacokinetics, Hemostatic Efficacy, and Safety of a New Human Fibrinogen Concentrate in Adult and Pediatric Patients with Congenital Fibrinogen Deficiency

**DOI:** 10.1055/a-2715-2994

**Published:** 2025-10-17

**Authors:** Claudia Djambas Khayat, Amal El-Beshlawy, Balkis Meddeb, Abderrahim Khelif, Wolfgang Miesbach, Sonia Adolf, Heike Boehm, Silke Aigner, Salomon Abraha, Fabian Bohlaender, Joerg Schuettrumpf

**Affiliations:** 1 Department of Pediatrics, Hotel Dieu de France Hospital Beirut, Saint Joseph University, Lebanon; 2 Pediatric Hospital CU, Egyptian Thalassemia Association, El Cairo, Egypt; 3 Hospital Aziza Othmana, Tunis, Tunisia; 4 Hospital Farhat Hached, Faculte de Medecine Ibn El Jazzar, Sousse University, Tunisia; 5 Department of Haemostasis/Haemophilia Centre, Medical Clinic 2, University Hospital Frankfurt, Frankfurt, Germany; 6 National Research Center, Pediatric Hematology Department, El Cairo, Egypt; 7 Biotest AG, Dreieich, Germany; 8 Grifols, Barcelona, Spain

**Keywords:** afibrinogenemia, hypofibrinogenemia, congenital fibrinogen deficiency, fibrinogen concentrate, pharmacokinetics

## Abstract

**ABSTRACT:**

**Background:**

Congenital fibrinogen deficiencies are rare coagulopathies which are treated by fibrinogen concentrates. This trial investigated the pharmacokinetic/pharmacodynamic (PK/PD) parameters, and surrogate efficacy and safety of a new human fibrinogen concentrate (HFC), BT524, in patients with congenital afibrinogenemia or severe hypofibrinogenemia.

**Methods:**

This prospective, multi-national, open-label, single-arm PK/PD trial evaluated PK/PD parameters of HFC (part 1; phase I) and HFC as on-demand treatment or prophylaxis for bleeding events (part 2; phase III). In part 1, patients received a single-dose of HFC (70 mg/kg body weight [BW]). PK/PD parameters were calculated using a PK/PD model and non-compartmental analysis. Fibrinogen antigen (FiAg) levels were determined over 14 days by immunonephelometry and fibrinogen activity (FiAc) by Clauss assay. The efficacy variable was mean change in maximum clot firmness (MCF) analyzed by thromboelastometry. Safety parameters were evaluated for 49 days.

**Results:**

A total of 27 patients ( *n* = 15 adults, *n* = 12 children) were treated with HFC. For FiAg, mean (SD) PK parameters were: C _max_ 1.81 (0.42) g/L, AUC _0-∞_ 173 (45.4) g*h/L, and t _1/2_ 67.9 (15.3) h. For FiAc, they were C _max_ 1.26 (0.4) g/L, AUC _0-∞_ 104 (33.5) g*h/L, and t _1/2_ 60.3 (13.3) h. In adults, MCF significantly increased 1 h after HFC infusion (11.1 (5.1) mm; *P* < 0.0001; 95% CI: 9.33–14.47). In pediatrics, mean increase in range was 9.3 to 16.5 mm. Treatment-related adverse events were rare, with one mild increase in fibrin D-dimer. No thromboembolic events, hypersensitivity, or allergic reactions were observed.

**Conclusion:**

HFC effectively increased FiAg levels and FiAc, improved clot firmness, and showed a favorable safety and tolerability profile in adult and pediatric patients with congenital fibrinogen deficiency.

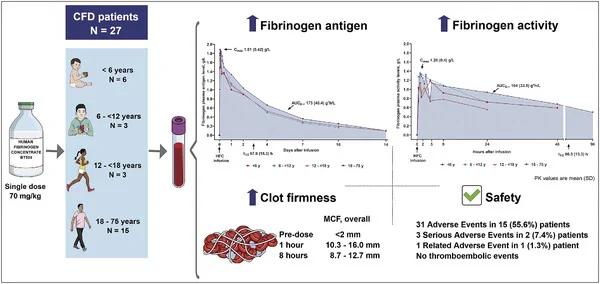

## Introduction

Fibrinogen (coagulation factor I) is one of the most abundant plasma proteins, with normal levels ranging from 2 to 4 g/L. 1 It plays several roles in maintaining hemostasis. In primary hemostasis, fibrinogen has an important role in mediating the binding among platelets through the glycoprotein GPIIb-IIIa, the central platelet receptor in platelet aggregation. 2 In secondary hemostasis, during the final step of the coagulation cascade, thrombin cleaves fibrinogen to fibrin. Fibrin molecules polymerize to form a three-dimensional network, and crosslinks introduced by factor XIII further stabilize the clot. 3 Beyond hemostasis, fibrinogen plays an important role in wound healing and host defense against microbes. 2

Congenital fibrinogen disorders are classified based on both clinical phenotypes and fibrinogen levels into four types: afibrinogenemia, hypofibrinogenemia, dysfibrinogenemia, and hypodysfibrinogenemia. 4 Quantitative disorders are characterized by a proportional reduction (hypofibrinogenemia) or absence (afibrinogenemia) of both functional fibrinogen and antigen concentration. Hypofibrinogenemia is classified according to the level of functional fibrinogen as severe (<0.5 g/L), moderate (0.5–0.9 g/L), or mild (≥1 g/L). 4 Dysfibrinogenemia and hypodysfibrinogenemia are qualitative disorders in which a disproportional reduction of functional fibrinogen compared to fibrinogen antigen is observed. 5 These types can be differentiated by assessing functional fibrinogen activity (FiAc), measured by clotting assays such as Clauss assay, and fibrinogen antigen (FiAg), measured by immunoassays such as ELISA, and immunonephelometry.

Afibrinogenemia is a rare disease 6 with a worldwide estimated prevalence of one to two patients per million, 7 but can be higher in populations with high rates of consanguinity. 8 The clinical phenotype of afibrinogenemia is heterogeneous and can lead to severe, even life-threatening bleeding, impaired wound healing, miscarriage, spleen rupture, and an increased risk of thrombosis in a subset of patients. 5 9 10 The most frequent manifestations are mucosal bleeding, menorrhagia, and musculoskeletal bleeding. 7 Hypofibrinogenemia can be asymptomatic but the clinical spectrum can also include bleeding and thrombosis. 7

Treatment of congenital fibrinogen deficiencies consists of supplementation of fibrinogen using fresh frozen plasma (FFP), cryoprecipitate, or fibrinogen concentrates. 11 The main limitation of using FFP or cryoprecipitate is that these products have non-standardized fibrinogen content and require larger volumes to achieve sufficient fibrinogen levels and hemostasis, particularly with FFP. Another limitation is the potential for transfusion-related risks such as the transmission of infectious agents, transfusion-related acute lung injury, or transfusion-associated circulatory overload. 12 13 As a result, fibrinogen concentrates are the current standard of care for fibrinogen replacement. 7 14 Although it should be noted that the availability of fibrinogen concentrates in low- and middle-income countries is lower. 15 Target plasma levels of fibrinogen are 1 g/L for minor and 1.5 g/L for major bleeding. 13 Based on data from a European registry, a level of >1 g/L is required to keep patients asymptomatic. 16 Fibrinogen concentrates have been shown to be safe, well-tolerated, and will rapidly restore fibrinogen levels to maintain hemostasis. 17 18

There is a need for prospective data with new fibrinogen concentrates. This trial evaluated a new human fibrinogen concentrate (HFC) in a clinical development program, which included the characterization of pharmacokinetic (PK) profile, and assessment of the efficacy and safety of the product. Therefore, the aim of this trial was to investigate the single-dose PK/PD, efficacy, and safety of fibrinogen concentrate in patients with congenital afibrinogenemia or severe congenital hypofibrinogenemia.

## Materials and Methods

### Trial Design and Objectives

This was a prospective, open-label, multinational, multicenter, phase I/III trial (NCT02065882). Pharmacokinetics/pharmacodynamics (PK/PD), surrogate efficacy, and safety of an HFC was investigated in patients with congenital fibrinogen deficiency, type I (afibrinogenemia, severe hypofibrinogenemia). Between March 2013 and May 2020, six active sites participated in the trial: one site each in Lebanon, Egypt, Bulgaria, and Germany, and two sites in Tunisia. Here, we report the results of the first part (phase I) in patients with congenital fibrinogen deficiency.

The primary objective was to determine the 14-day single-dose PK of HFC following IV infusion in patients with congenital afibrinogenemia or severe congenital hypofibrinogenemia. Secondary objectives were to investigate the 14-day single-dose PD, surrogate efficacy, and safety of a single administration of HFC.

The trial was conducted in accordance with applicable regulatory requirements, the International Conference of Harmonization Good Clinical Practice guidelines, the Declaration of Helsinki (version 17th, 2013), and local laws and regulations. 19 The trial was approved by the responsible independent ethics committees and regulatory authorities. Written informed consent to participate in the trial was obtained from each patient or, in case of pediatric patients, from their parents or legal representative, as applicable.

### Human Fibrinogen Concentrate

The investigational product evaluated in this trial was BT524, a lyophilized, heat-treated, HFC manufactured from human plasma (Biotest AG, Dreieich, Germany). It has a high safety margin for the risk of viral or prion transmission. Three viral inactivation and removal steps are included in the manufacturing process to ensure safety. Each vial contains 1 g fibrinogen, measured by fibrinogen activity assay, which is reconstituted with 50 mL of water for injection. Final concentration after reconstitution is 1 g/50 mL.

### Patients

Male or female patients aged 0 to 75 years with congenital afibrinogenemia or severe hypofibrinogenemia and documented fibrinogen levels of ≤0.5 g/L for both FiAc and FiAg were eligible for inclusion in the trial.

Main exclusion criteria were bleeding disorders other than congenital fibrinogen deficiency, history of esophageal variceal bleeding, thrombosis within 6 months before enrollment, hypersensitivity to fibrinogen or other plasma products, inhibitory antibodies to fibrinogen, treatment with fibrinogen containing product or concomitant medication relevantly interacting with the coagulation system within 2 weeks, and vaccination within 3 weeks before infusion of HFC. In addition, patients were also excluded if they had body weight ≤22 kg for patients ≥6 years, and ≤5th percentile of normal range for children <6 years, 20 end stage disease, pregnancy, infection, positive serology for HIV, or active or anticipated bleeding.

An independent data monitoring committee was established to ensure safety in this first-in-human administration of HFC. Children ≥6 years and adolescents were only enrolled after 10 adults completed dosing of HFC and a 7-day safety follow-up period. Children <6 years were enrolled only after data from the rest of patients were reviewed.

### Dosing and Assessments

Each patient received a fixed intravenous dose of 70 mg per kg BW of HFC. Dosing was based on published PK data and information from comparator preparation 21 and confirmed by preclinical studies. It was calculated according to the measured activity of fibrinogen in each batch used. Infusion rate was 5 mL/min (100 mg/min) for all patients ≥6 years of age, while lower rates were selected for children <6 years of age at the discretion of the investigator.

### Pharmacokinetics and PK/PD Model

In trial participants 6 years old and older and BW >43 kg, samples for PK analysis (FiAc and FiAg) were obtained pre-dose, post-infusion, and 30 min, 1, 2, 4, and 8 hours and 1, 2, 4, 7, 10, and 14 days post-infusion. For children ≥6 years old with BW ≤43 kg, a reduced PK sampling schedule included pre-dose, post-infusion, 1 and 4 hours, and 1, 4, 7, and 10 days post-infusion. In all children under 6 years of age, two samples were consistently collected at the same time points: at pre-dose and post-infusion. In children aged 2 to <6 years, three additional samples were drawn: at 1 and 12 hours and 2 days post-infusion. In younger children (<2 years) a reduced sampling schedule was followed with samples taken at 4 hours and 1 day post-infusion at the discretion of the investigator.

Plasma samples were analyzed at University of Duesseldorf (Institute of Hemostaseology, Hemotherapy and Transfusion Medicine, Dusseldorf, Germany) for FiAg level and FiAc. FiAg was determined by immunonephelometry (using Siemens BN ProSpec Analyzer), with a detection limit of 0.03 mg/L and a lower limit of quantification (LLOQ) of 0.1 g/L. FiAc was measured by Clauss assay (Siemens BCS XP Analyzer, Siemens Healthineers AG, Forchheim, Germany), with a detection limit of 0.2 g/L and a LLOQ of 0.35 g/L.

The primary PK endpoints were the parameters derived from time concentration profiles using adapted methodology (non-compartmental analysis, NCA), compartmental analysis, or population modeling. A population PK/PD model was developed to simulate dense FiAg and FiAc concentration versus time profiles. All data from adults and children were used in a stepwise approach. The PK characteristics of FiAg after IV administration of HFC were well described by a two compartmental disposition model with first-order elimination, and the relationship between the FiAg and FiAc was described by a proportional model. Performance of the model was evaluated by internal validation versus trial data (visual predictive checks) and externally by comparison with historical data. 21

Age, sex, and BW were tested as covariates. The stepwise covariate model building procedure did not identify any significant covariates–parameter relationships. The model was used to predict full individual profiles for infants and newborns with sparse or reduced PK/PD data sets.

The NCA simulated data of individual FiAg and FiAc concentration time profiles, after dosing 27 patients (15 adults aged 18 to 75 years, 3 adolescents aged 12 to <18 years, 3 children aged 6 to <12 years, and 6 children aged <6 years). The simulated profiles were derived using the empirical Bayes estimates from the population PK and PKPD analyses and the dosing and demographic data. The simulated concentration/time profiles were used within an NCA to obtain the individual PK/PD parameters: maximum plasma concentration (C _max_ ), area under the concentration–time curve from time 0 to infinity (AUC _0-∞_ ), half-life (t _1/2_ ), mean residence time (MRT), incremental and classical in vivo recovery (IVR), volume of distribution at presumed steady-state (Vd _ss_ ) and clearance (CL).

### Surrogate Efficacy

The efficacy variable was maximum clot firmness (MCF) measured 1 and 8 hours after HFC infusion. MCF was determined by means of the Fib-tem S assay (tissue factor activation and platelet inhibition), a ready-to-use thromboelastometry system reagent, which allows the assessment of fibrinogen levels and the quality of fibrin polymerization in citrated blood by inhibiting the platelets. 22 MCF was measured by thromboelastometry (ROTEM device, Tem International GmbH, Munich, Germany) on citrated plasma samples obtained at pre-dose and at 1 and 8 hours post-infusion. In children <6 years, only two measurements were assessed: pre and 1 hour post-infusion. MCF was quantified as surrogate marker for hemostatic efficacy as previously described. 23 For thromboelastometry, the coagulation of citrated blood sample was activated by different reagents, depending on the assay performed. Various clot properties during formation and lysis of the clot can be determined. The test was performed on frozen plasma samples instead of whole blood, using the FIBTEM-validated assay at the central laboratory. In FIBTEM analysis, coagulation is activated by a small amount of tissue thromboplastin and thrombocytes are blocked with cytochalasin D. The clot is then dependent only on fibrin formation and polymerization. Reference range for all age groups were 9 to 25 mm, and the lower limit of detection was 2 mm. Values below the lower limit of detection were documented as “not measurable” by the central laboratory and they were set to half of the range below the detection limit. The range was between 0 and 2 mm; therefore, the value was set as 1 mm for the purpose of the planned descriptive statistical analyses.

### Safety

Safety analysis included the evaluation of all adverse events (AEs) and serious adverse events (SAEs) that occurred or worsened during or after the first HFC administration until the last visit (day 49 [ ± 4 days]) (treatment-emergent AEs/SAEs). AEs were collected throughout the trial period and classified by seriousness (serious or not serious), causality (related or not related to the trial medication), and severity. The severity of AEs was categorized as mild, if it did not interfere with daily activities, moderate if it interfered with the patient's daily routine, but allowed continuation of usual routine, and severe if it prevented the performance of routine activities. AEs were coded using Medical Dictionary for Regulatory Activities, MedDRA, version 23.0. Predetermined adverse events of special interest (AESI) included hypersensitivity, anaphylactic reactions including shock, embolic and thrombotic events, transmission of infectious agents, and fibrinogen inhibitory antibodies. Determination of antibodies directed against fibrinogen epitopes leading to inhibition of FiAc was performed at the screening visit and at the last visit.

Safety was also evaluated based on clinical laboratory parameters: hematology, clinical chemistry, urinalysis, and coagulation markers including prothrombin time (PT), activated partial thromboplastin time (aPTT), thrombin–antithrombin-III complex (TAT), prothrombin fragment 1 and 2 (F1 + 2), and D-dimer. Additionally, vital signs, physical examinations, and virus status were summarized from HFC administration until the last visit.

### Statistical Analysis

For the PK parameters, no formal sample size calculation was carried out. As a general rule approximately 20 patients in this single cohort should suffice to calculate PK parameters with the corresponding range of distribution. 24

A total of four different populations were analyzed. The intention to treat (ITT), which was identical to the safety analysis set (SAF), included all patients who received fibrinogen concentrate. The full-analysis set (FAS) included all patients of the ITT with at least one available efficacy assessment. The per-protocol (PP) set was defined as all patients without major protocol deviations with potential impact on PK/PD results. The PK analysis set included all patients with PK data.

Descriptive statistics were used to summarize PK/PD, efficacy, demographic, and safety parameters. The mean change in MCF between pre-dose and 1 hour post end of infusion was tested using a two-sided t-test (α-level 0.05); 95% confidence interval (CI) was calculated. Spearman's correlation coefficients were used between MCF and FiAc pre-dose, 1 and 8 hour post-infusion. Safety analysis was based on the SAF population. *P* values <0.05 were considered statistically significant. SAS version 9.4 (SAS Institute, Cary, North Carolina, United States of America) was used for statistical analysis calculations.

## Results

### Trial Population—Patient Characteristics

**Fig. 1:**
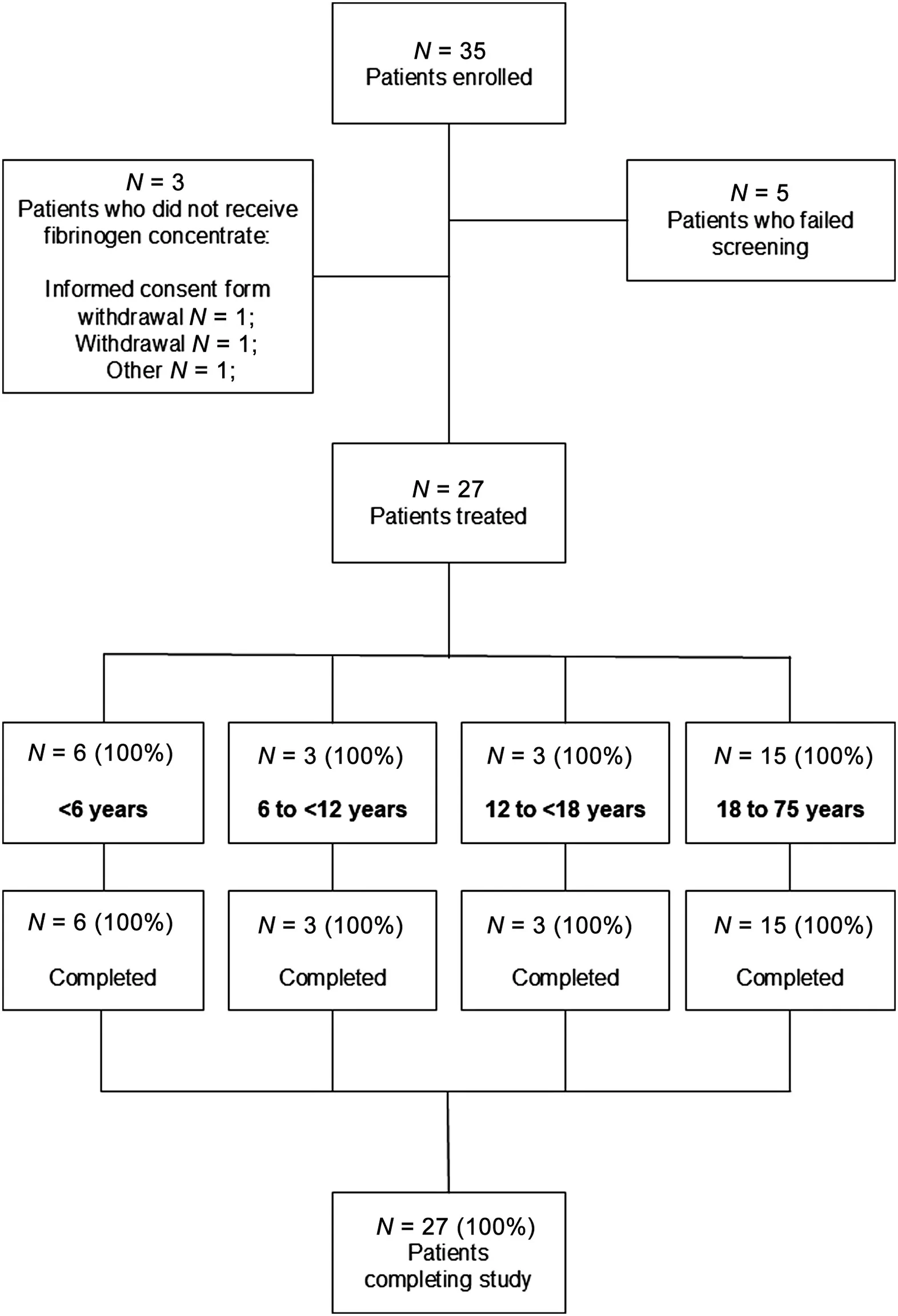
Flowchart of patients through the trial.

A total of 35 patients were enrolled in the PK/PD part of the trial. There were 5 screening failures and only 30 patients were eligible for treatment. Three patients withdrew; therefore, 27 patients were treated with HFC, included in the PK/PD analysis, and completed the trial. Of those, 15 were adults, 3 each were 12 to <18 years and 6 to <12 years old, and 6 patients were <6 years old. Disposition of patients included in the trial is summarized in Fig. 1 . Demographics and baseline characteristics of patients included in the trial, by age group, are shown in Table 1 . All 27 patients were diagnosed with congenital afibrinogenemia. There were 14 males (51.9%) and 13 females (48.1%). Concomitant medications reported were analgesics in eight (29.6%) patients, and antianemia preparations (iron) and antibacterials for systemic use in three (11.1%) patients each. The number of patients recruited per center is shown in Supplementary Table S1 (available in the online version only).

**Table 1:** Demographics and other baseline characteristics of patients

	<6 years, *n* = 6	6 to <12 years, *n* = 3	12 to <18 years, *n* = 3	18 to 75 years, *n* = 15	Overall, *n* = 27
Sex, male, *n* (%)	2 (33.3)	0 (0)	1 (33.3%)	11 (73.3)	14 (51.9)
Female	4 (66.7)	3 (100)	2 (66.7)	4 (26.7)	13 (48.1)
Race					
White	6 (100)	2 (66.7)	3 (100)	15 (100)	26 (96.3)
Other	0 (0)	1 (33.3)	0 (0)	0 (0)	1 (3.7)
Age, y, mean (SD)	2.2 (1.6)	8.7 (2.5)	13.3 (1.2)	26.4 (7.2)	17.6 (11.8)
Weight, kg, mean (SD)	13.52 (2.9)	32.33 (11.1)	46.33 (7.4)	71.87 (14.0)	51.7 (27.2)
BMI, kg/m ^2^ , mean (SD)	17.3 (1.4)	17.6 (4.2)	18.7 (4.2)	25.7 (5.1)	22.1 (5.8)
Disease characteristics					
Afibrinogenemia	6 (100)	3 (100)	3 (100)	15 (100)	27 (100)
Frequency of historic bleeding events, *n* (%) ^a^					
Umbilical cord bleeding	6 (100)	3 (100)	3 (100)	12 (80)	24 (88.9)
Oral cavity bleeding	2 (33.3)	3 (100)	1 (33.3)	14 (93.3)	20 (74.1)
Muscle hematoma	1 (16.7)	1 (33.3)	1 (33.3)	11 (73.3)	14 (51.9)
Epistaxis	2 (33.3)	1 (33.3)	1 (33.3)	10 (66.7)	14 (51.9)
Hemarthrosis	0 (0)	0 (0)	1 (33.3)	12 (80)	13 (48.1)
Intraperitoneal bleeding	0 (0)	1 (33.3)	1 (33.3)	5 (33.3)	7 (25.9)
Other	4 (66.7)	1 (33.3)	3 (100)	11 (73.3)	19 (70.4)
Annual bleeding rate b , mean (SD)	1.8 (0.98)	1	2	2 (1.63)	1.9 (1.37)

Abbreviations: BMI, body mass index; SD, standard deviation; y, years.

a Frequency of historic bleeding events in ≥20% of patients in the overall group.

b Number of bleedings within the last 12 months before informed consent form signature. The number of patients for each group was: <6 years, *n* = 6; 6 to <12 years, *n* = 1; 12 to <18 years, *n* = 1; 18 to 75 years, *n* = 13.

Mean (SD) treatment duration was 56.6 (10.96) min in adults, whereas in the pediatric groups it was shorter: 34.7 (4.37) min, <6 years; 25.3 (7.02) min, 6 to <12 years; and 37.3 (6.81) min, 12 to <18 years. Infusion rates were 4.98 (0.30) mL/min in adults, 1.39 (0.21) mL/min in <6 years old, and 5 mL/min in 6 to <12 years old and 12 to <18 years old groups. There were no changes in infusion rates in any patient.

### PK/PD Characteristics

**Fig. 2:**
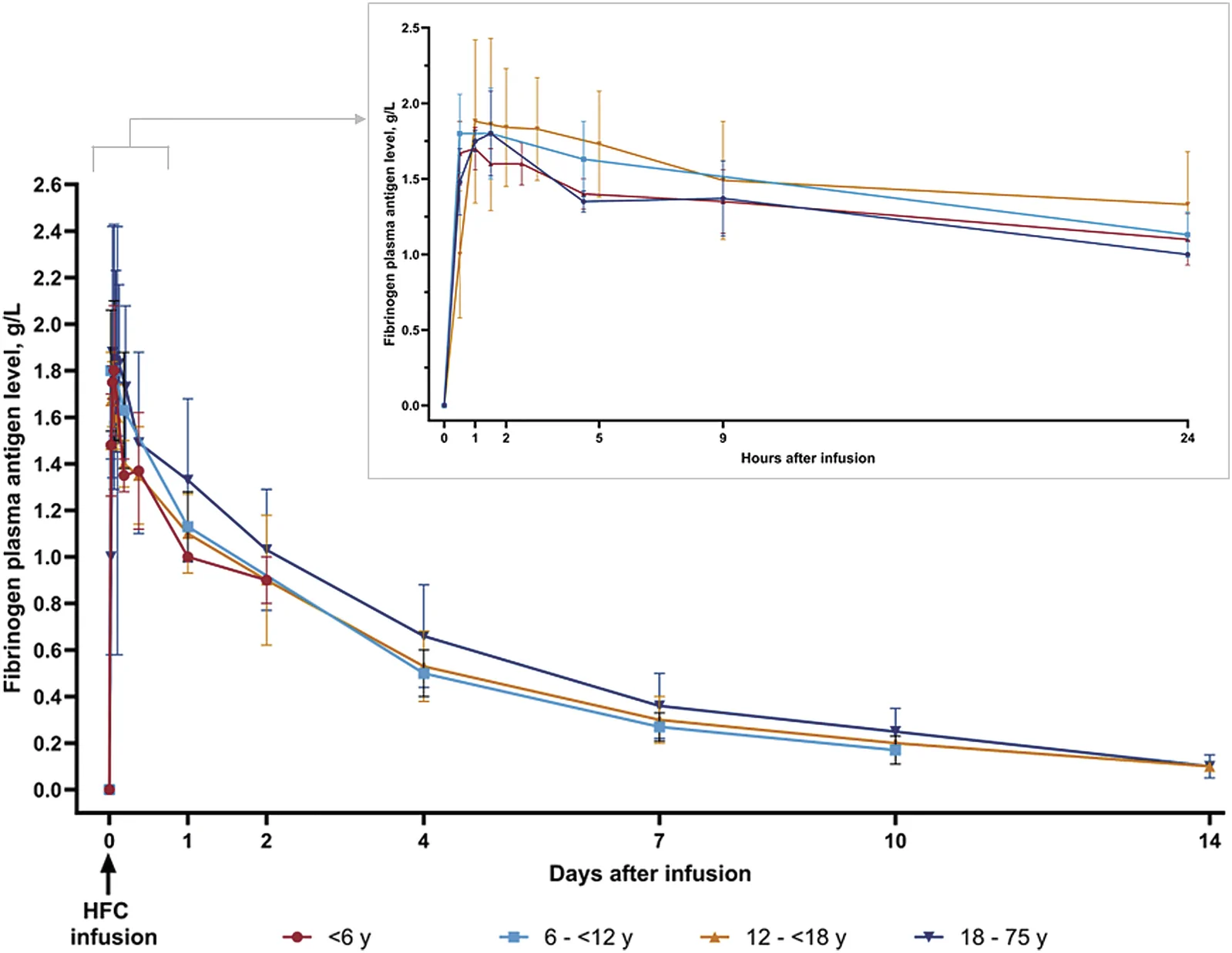
Mean (SD) fibrinogen antigen levels in plasma over time (PK) by age group. Lines represent the mean of the observed FiAg profiles at planned time points. Data points with at least two patients are represented. The number of available patient samples varied across time points as follows: <6 years, 2 to 4 patients; 6 to <12 years, 3 patients; 12 to <18 years, 2 or 3 patients; 18 to 75 years, 12 to 15 patients.

**Fig. 3:**
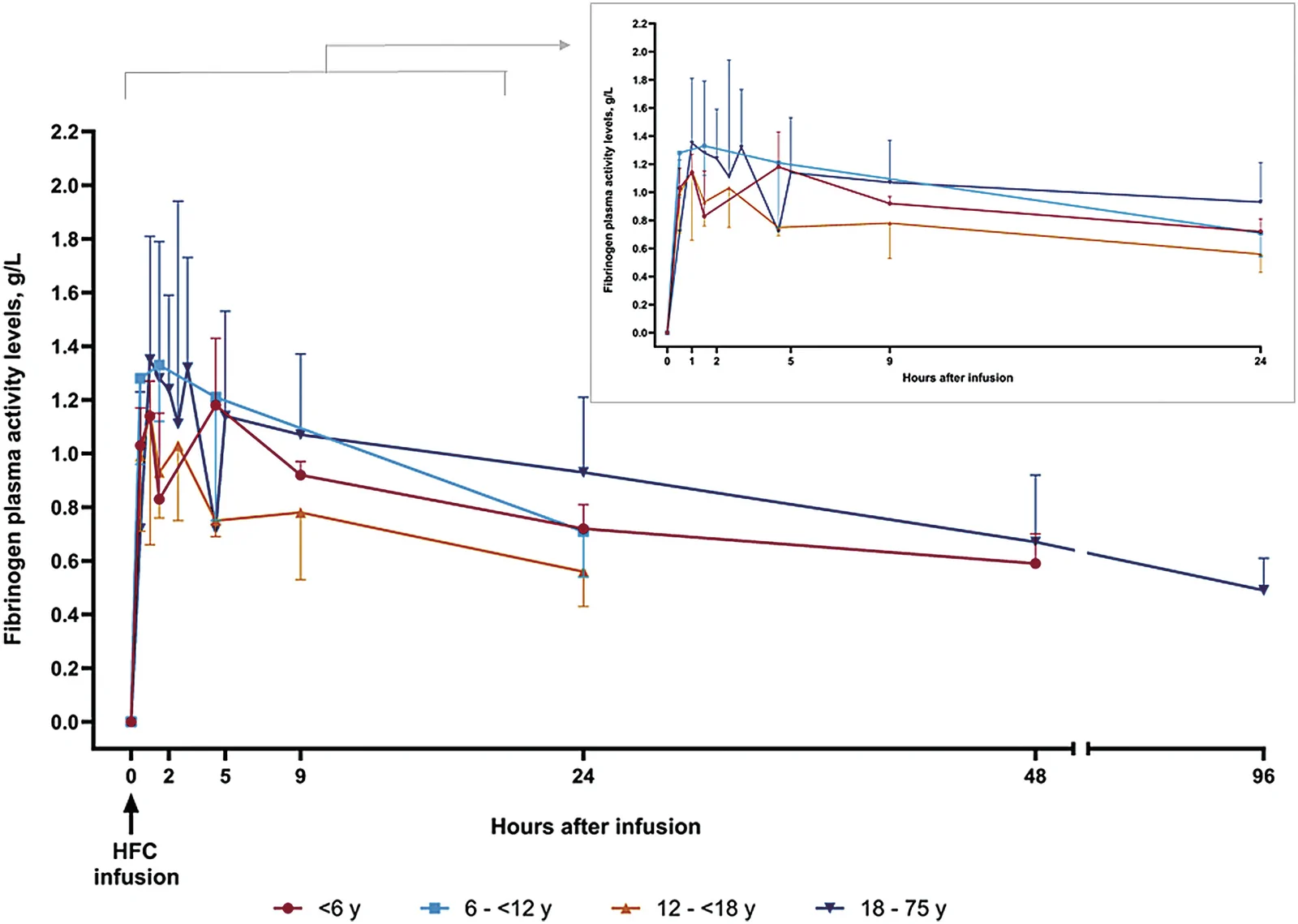
Mean (SD) fibrinogen activity (FiAc) levels in plasma over time (PD) during the first 24 hours after human fibrinogen concentrate (HFC) administration and during 4 days after HFC by age group. Data points with at least two patients are represented. The number of available patient samples varied across time points as follows: <6 years, 2 to 4 patients; 6 to <12 years, 3 patients; 12 to <18 years, 2 or 3 patients; 18 to 75 years, 8 to 14 patients.

The PK (FiAg levels) and PD (FiAc) of HFC were evaluated after single administration of 70 mg/kg BW. HFC administration resulted in a rapid increase in mean FiAg values from 0 g/L at pre-dose up to 1.86 g/L at post-infusion. It was followed by a slow decline of FiAg reaching baseline values 14 days after injection ( Fig. 2 ). A similar profile was observed for FiAc ( Fig. 3 ). There were no differences between age groups.

To determine PK parameters and to get a deeper understanding of the PK characteristics of HFC, especially in groups with sparse sampling (e.g., young children), a population PK/PD model was developed. The model was used to simulate dense FiAg and FiAc concentration versus time profiles for patients with sparse data and to investigate the influence of covariates on PK. The performance of the final population PK and PK/PD was evaluated using internal as well as external validation and showed that the model provided a good description of the data the model was developed on. The model simulated concentrations similar to the ones observed in the trial across all age groups. Neither age, sex, nor BW could be identified as significant covariates.

The non-compartmental analysis for determination of HFC PK parameters was based on the simulated individual FiAg and FiAc concentration versus time profiles. PK parameters for FiAg and FiAc were derived for the overall trial population and for each age group ( Table 2 ).

**Table 2:** PK/PD parameters for fibrinogen antigen (FiAg) and fibrinogen activity (FiAc) by age group

	<6 years, *n* = 6	6 to <12 years, *n* = 3	12 to <18 years, *n* = 3	18 to 75 years, *n* = 15	Overall, *n* = 27
FiAg (g/L), pre-dose mean (SD)	0.02 (0.041)	0.03 (0.058)	0.03 (0.058)	0.02 (0.0442)	n/a
t _1/2_ (h)	52.4 (4.81)	58.2 (5.25)	67.4 (8.27)	76.2 (14.6)	67.9 (15.3)
C _max_ (g/L)	1.69 (0.177)	1.89 (0.295)	1.68 (0.15)	1.87 (0.54)	1.81 (0.42)
AUC _0-∞_ (g × h/L)	145 (16.1)	153 (11.3)	156 (28.4)	191 (52.6)	173 (45.4)
MRT _0-∞_ (h)	143 (19.3)	122 (20)	128 (14.7)	132 (16.4)	133 (17.4)
Vd _ss_ (mL/kg)	70.4 (15.8)	56.5 (12.8)	59.0 (14.4)	52.9 (21)	57.8 (19.1)
CL (mL/h/kg)	0.488 (0.049)	0.459 (0.033)	0.459 (0.076)	0.395 (0.116)	0.43 (0.995)
IR _obs_ (mg/dL per mg/kg dose)	2.45 (0.291)	2.62 (0.438)	2.43 (0.242)	2.74 (0.83)	2.63 (0.652)
FiAc (g/L), pre-dose mean (SD)	0.150 (0)	0.175 (0)	0.175 (0)	0.175 (0)	n/a
t _1/2_ (h)	46.9 (3.96)	51.9 (4.96)	60.5 (6.53)	67.4 (12.9)	60.3 (13.3)
C _max_ (g/L)	1.16 (0.125)	1.32 (0.279)	1.04 (0.194)	1.33 (0.499)	1.26 (0.396)
AUC _0-∞_ (g × h/L)	86.7 (7.6)	93.8 (12.6)	84.8 (23.1)	116 (39.6)	104 (33.5)
MRT _0-∞_ (h)	134 (18)	120 (11)	119 (14.9)	122 (16.4)	124 (16.2)
Vd _ss_ (mL/kg)	110 (22.3)	91.1 (19.6)	103 (30.1)	83.6 (40.5)	92.4 (34.7)
CL (mL/h/kg)	0.814 (0.075)	0.755 (0.097)	0.863 (0.205)	0.671 (0.238)	0.733 (0.203)
IR _obs_ (mg/dL per mg/kg dose)	1.6 (0.201)	2.04 (0.466)	1.59 (0.494)	2.03 (0.729)	1.88 (0.61)

Abbreviations: AUC _0-∞_ , area under the curve from time 0 to infinity; C _max_ , maximum concentration; CL, clearance; IRobs, incremental recovery calculated from observed values MRT _0-∞_ , mean residence time extrapolated to ∞; *n* , number of patients; t _1/2_ , half-life; Vd _ss_ , volume of distribution at presumed steady-state; y, years.

In addition to Table 2 , which shows mean values, the geometric mean of incremental recovery (IR) for FiAc was 1.78 mg/dL per mg/kg dose in the overall group, 1.87 mg/dL per mg/kg dose in adults, 1.54 mg/dL per mg/kg dose in the 12 to 18 years old group, 2.00 mg/dL per mg/kg dose in the 6 to 12 years old group, and 1.59 mg/dL per mg/kg dose in the <6 years old group. Geometric mean values of IR were used for the calculation of the pediatric dose.

Overall, these data demonstrated that the administration of HFC resulted in an increase of systemic fibrinogen concentration (FiAg) in patients, which was associated with an enhanced fibrinogen activity (FiAc). The results did not suggest remarkable differences between adults and children in the exposure and PK properties of the HFC administered intravenously.

### Surrogate Efficacy

**Fig. 4:**
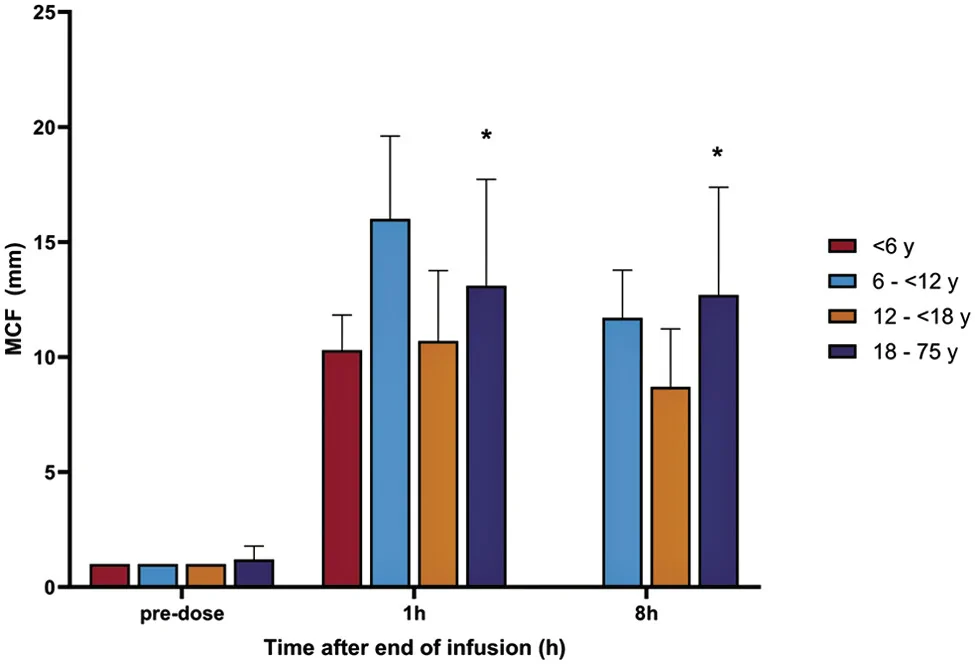
Mean (SD) FIBTEM maximum clot firmness (MCF) over time (pre-dose, 1 h and 8 h after end of fibrinogen concentrate infusion). The number of patients were: for the <6 years old group, 6 patients at pre-dose and 3 patients at 1 h; for the 6 to <12 years and 12 to <18 years old groups, 2 patients at pre-dose, 3 patients at 1 h, and 3 patients at 8 h post-infusion for each group; for the 18 to 75 years old group, 12 patients at pre-dose, 15 patients at 1 h, and 14 patients at 8 h post-infusion. T test was not performed for groups with less than five patients (<6, 6–12, and 12–18 years). Lower limit of detection was 2 mm. Values below this limit were reported as 1 mm. * *p* -value <0.001 at 1 h and at 8 h versus pre-dose.

Patients had FIBTEM MCF values below the detection limit (2 mm) before they were treated with HFC. After infusion of HFC, MCF increased considerably reaching mean values of 10.3 to 16.0 mm in the different age groups at 1 hour post-infusion. At 1 hour after infusion of HFC, MCF reached mean (SD) values of 10.3 (1.53) mm in children under 6 years, 16.0 (3.61) mm in children 6 to 12 years, 10.7 (3.06) mm in adolescents aged 12 to 18 years, and 13.11 (4.62) mm in adults. In adults, the MCF increase was statistically significant at 1 hour post-infusion compared to pre-infusion values (11.1 (5.07) mm; *p* < 0.0001, 95% CI [9.33; 14.47]). At 8 hour post infusion, the increase in FIBTEM MCF was sustained and started to decrease slightly, with mean values between 8.7 and 12.7 mm. The difference between pre-dose and 8 hours post-infusion MCF was statistically significant in adults (11.3 (5.08) mm; *p* < 0.0001, 95%CI 8.83–14.26). Statistical analysis was not conducted for groups with less than five patients (<6, 6–12, and 12–18 years) ( Fig. 4 ).

FIBTEM MCF showed a high correlation with FiAc levels at 1 hour after post-infusion of HFC: r = 0.80 in the FAS population and r = 0.70 in the PP population, across all age groups. Similarly, a strong correlation was observed between MCF and FiAc levels 8 hours post-infusion of HFC (FAS, r = 0.80; PP: 0.72).

### Safety

Overall, 31 AEs were reported in 15 (55.6%) patients. The most frequent AEs by MedDRA SOC were seven gastrointestinal disorders experienced by four (14.8%) patients, followed by five infections and infestations experienced by five (18.5%) patients. Most AEs occurred as single events reported for individual patients across all age groups. No thromboembolic events, hypersensitivity, anaphylactic reactions, or serologic conversions were reported. Most of the AEs were either mild or moderate. Severe events were only experienced by two (7.4%) patients, all assessed as not related to HFC. Notably, only one mild AE, increased fibrin D-dimer, was assessed as related to HFC treatment ( Table 3 ).

**Table 3:** Summary of adverse events (AEs) in the safety analysis set

	<6 years, *n* = 6		6 to <12 years, *n* = 3		12 to <18 years, *n* = 3		18 to 75 years, *n* = 15		Overall, *n* = 27	
	Events	Patients (%)	Events	Patients (%)	Events	Patients (%)	Events	Patients (%)	Events	Patients (%)
AEs	1	1 (16.7)	3	2 (66.7)	3	2 (66.7)	24	10 (66.7)	31	15 (55.6)
Related AEs	0	0 (0)	0	0 (0)	0	0 (0)	1	1 (6.7)	1	1 (3.7)
Severe AEs	0	0 (0)	0	0 (0)	2	1 (33.3)	3	1 (6.7)	5	2 (7.4)
SAEs	0	0 (0)	0	0 (0)	2	1 (33.3)	1	1 (6.7)	3	2 (7.4)
Related SAEs	0	0 (0)	0	0 (0)	0	0 (0)	0	0 (0)	0	0 (0)
AEs leading to trial discontinuation	0	0 (0)	0	0 (0)	0	0 (0)	1	1 (6.7)	1	1 (3.7)
AEs leading to death	0	0 (0)	0	0 (0)	0	0 (0)	0	0 (0)	0	0 (0)
AEs by SOC/PT a										
Gastrointestinal disorders	0	0 (0)	1	1 (33.3)	0	0 (0)	6	3 (20)	7	4 (14.8)
Toothache	0	0 (0)	0	0 (0)	0	0 (0)	2	2 (13.3)	2	2 (7.4)
Musculoskeletal	0	0 (0)	0	0 (0)	1	1 (33.3)	4	2 (13.3)	5	3 (11.1)
Back pain	0	0 (0)	0	0 (0)	1	1 (33.3)	3	2 (13.3)	4	3 (11.1)

Abbreviations: AE, adverse event; SAE, serious adverse event; SOC, system organ class; PT, preferred term.

Notes: Safety analysis included the evaluation of treatment-emergent adverse events (TEAEs).

a AEs by SOC/PT: occurrence in >5% of patients in the overall group.

Three SAEs (pain in left toe, post-streptococcal glomerulonephritis, hypertensive encephalopathy) were reported in two (7.4%) patients. One AE in one (3.7%) patient, pain in extremity, led to trial discontinuation. No antibodies to fibrinogen were detected in any patient at the safety follow-up visit on day 49.

Values for hematological parameters and clinical chemistry did not show clinically relevant changes during the trial. For coagulation parameters, aPTT and PT decreased 1 day after administration of HFC and gradually increased over time again to pre-dose levels throughout the study period. Results for TAT, F1 + 2, and D-dimer are shown in Supplementary Table S2 (available in the online version only). Remarkably, D-dimers remained stable and low across all time points during the study period.

## Discussion

This trial evaluated the PK/PD characteristics, surrogate efficacy, and safety of a single dose of an HFC in 15 adults and 12 children/adolescents with congenital fibrinogen deficiency, type I (afibrinogenemia, severe hypofibrinogenemia). This trial showed the PK/PD profile of this novel HFC in patients with congenital fibrinogen deficiencies. The HFC effectively increased FiAg and FiAc, restored clot formation, and demonstrated a favorable safety and tolerability profile across all age groups.

Patients were treated with a single intravenous dose of 70 mg/kg BW of HFC, the same dose used with other fibrinogen concentrates. 21 25 26 27 However, a lower dose of 60 mg/kg BW has been used for a different HFC product. 28 29 In our trial, HFC increased fibrinogen levels from below detection limit to a mean of 1.86 g/L at 1 hour after end of infusion. This result is consistent with peak fibrinogen concentrations previously reported in trials of the same indication with other fibrinogen concentrates. 21 25 26 27 30 31 This comparison among trials was considered acceptable despite certain limitations for these comparisons such as a small number of patients, and variations in laboratory assays and PK sampling procedures. It should be noted that there are no international standards to compare results between laboratories and assays. 21 26 WHO standards are available for the fibrinogen functional assay, 32 improving comparability of results obtained across different laboratories and assays. However, such standards do not exist for immunological methods to measure FiAg, 32 which limits the comparability between trials, even if identical methods are used. FiAc reached a C _max_ of 1.26 g/L. Although the FiAc was lower than FiAg, such results were also previously reported with other fibrinogen concentrates. 29 Importantly, the overall exposure to the product was like in these previous studies, 21 29 demonstrating that enough functionally active fibrinogen is supplied to the patient. The differences in C _max_ between FiAg and FiAc levels could also be explained by the different assays used for determination in plasma samples and potential variability in the assays. The purity of fibrinogen batches was determined based on total protein and fibrinogen activity, and had to be >80% to comply with European Pharmacopeia specifications. 33 The purity of the batches used in the current trial was 91 and 99%. Consequently, the differences between fibrinogen antigen and activity levels were considered minor.

After HFC infusion, differences in PK parameters have been previously reported when comparing adult and children populations. 25 26 30 Similar, a tendency of lower C _max_ in younger children, with a mean value of 1.16 g/L in children <6 years compared to adults with 1.33 g/L, was observed in the current trial. Similarly, AUC _0-∞_ was lower and t _½_ was shorter in children compared to adults, while clearance was higher, as reported in other trials. 26 28 34 The reduced AUC _0-∞_ and increasing elimination rates in children are an expected consequence of BW dosing, where the metabolic capacity per kilogram is known to increase with decreasing BW according to allometric principles. 35 Nonetheless, the small sample size of patients and the between-patients variability hinder the derivation of any substantial conclusions. The overall geometric mean incremental IVR of 1.78 mg/dL per mg/kg BW across all age groups was comparable to other trials conducted with the same dose of HFC, which ranged from 1.46 to 1.96 mg/dL. 26 30

Hemostatic efficacy of HFC was demonstrated by a statistically significant increase in FIBTEM MCF values ranging from 9.3 mm in children under 6 years to 16.5 mm in children between 6 and 12 years 1 hour after administration of HFC, and only declined slightly at 8 hours post administration. The FIBTEM MCF increase from baseline was similar to the increase observed with other fibrinogen concentrates. 21 26 27 29 More importantly, a high correlation was observed between FIBTEM MCF and fibrinogen levels, indicating that the restored fibrinogen was functional.

MCF was assessed using thromboelastometry on plasma samples instead of whole blood. Plasma has been suggested as an alternative to whole blood, especially in the research setting, to reduce variability in local testing. 36 Plasma samples have also been used in other trials with fibrinogen concentrate to determine MCF either by FIBTEM or EXTEM. 21 26 27 29 Of note, MCF reference range values of whole blood differ substantially between FIBTEM (6–19 mm) and EXTEM (57–72 mm). 37 As stated above for fibrinogen levels, comparison with other trials have some limitations, as differences were expected between different laboratories and assays, FIBTEM 21 28 29 versus EXTEM, 27 and sample type, plasma 21 27 28 29 versus whole blood. 26

In our trial, the HFC was well tolerated in adults and pediatric patients. HFC safety profile aligned with other fibrinogen concentrates and was consistent with the nature of the underlying disease. The majority of adverse events were mild or moderate in severity, as previously observed with other licensed fibrinogen concentrates. 27 38 39 Moreover, only one mild adverse reaction, an increase in D-dimer, was observed in the current trial. A recent safety analysis including post-marketing data and clinical trials confirmed a low rate of adverse reactions with fibrinogen concentrates. 40 Neutralizing antibodies following administration of HFC in patients with congenital afibrinogenemia seems to be rare, but case reports can be found in the literature. 6 41 In the current trial, no antibodies against fibrinogen were detected by day 49.

There are some limitations of the current trial. Since congenital fibrinogen deficiency is an ultra-rare disease with an estimated incidence of 1 in 1,000,000 live births worldwide, 42 43 a limited number of patients were enrolled. Of the 27 patients evaluated for PK/PD analysis, a small number of adolescents and children 6 to 12 years old were included. Although these data were substantiated by population PK/PD modeling, the comparison between age groups within this trial and the comparability to other trials are limited.

Congenital fibrinogen deficiency is associated with severe and/or frequent bleeding episodes for which fibrinogen supplementation is essential to prevent or treat bleeding events. 44 Following the completion of part 1 of this clinical trial (phase I), part 2 (phase III) was conducted to further evaluate the hemostatic efficacy of fibrinogen concentrate for the treatment and prophylaxis of bleeding events in patients with congenital fibrinogen deficiency. Further research on the use of fibrinogen concentrates for treating other types of fibrinogen deficiencies (e.g., acquired fibrinogen deficiency in patients undergoing major surgeries) is warranted.

In summary, the trial demonstrated that a single dose of 70 mg/kg BW HFC in patients with congenital afibrinogenemia effectively increased plasma levels of FiAg coupled with enhanced FiAc. Likewise, after HFC administration, FIBTEM MCF, as surrogate marker for efficacy, was increased, and coagulation assays aPTT and PT/INR improved. Additionally, HCF showed a favorable safety and tolerability profile, supporting its use in clinical practice. Furthermore, the PK/PD data demonstrate the suitability of the formulation for both pediatric and adult populations, as well as the appropriate use in all age groups using a BW-based dosing approach.

## Notes

**What is known about this topic?**

- Congenital fibrinogen deficiencies result from a quantitative or qualitative deficiency in fibrinogen levels.
- Fibrinogen concentrates are the treatment of choice in patients with congenital fibrinogen deficiencies.

**What does this paper add?**

- This trial assessed pharmacokinetics, efficacy, and safety of a human fibrinogen concentrate (HFC) in patients with congenital fibrinogen deficiencies.
- Treatment with HFC increased fibrinogen antigen levels and fibrinogen activity.
- Treatment with HFC demonstrated hemostatic efficacy and showed a favorable safety and tolerability profile.
