# Supplementary material for: Pharmacokinetics, Hemostatic Efficacy, and Safety of a New Human Fibrinogen Concentrate in Adult and Pediatric Patients with Congenital Fibrinogen Deficiency
Source: Thromb Haemost. 2025 Oct 17;126(9):919–29. doi: 10.1055/a-2715-2994 (PMC13521662; doi:10.1055/a-2715-2994)
Supplement: Supplementary file 1 [file 10-1055-a-2715-2994_29337250.pdf]

Thieme

Supplementary Material

Supplementary Table 1. List of patients enrolled and treated with human fibrinogen concentrate by site.

Site	Country, City	Patients enrolled, N=35	Patients treated, N=27
Site 01	Lebanon, Beirut	21	20
Site 11	Egypt, Cairo	8	3
Site 12	Tunisia, Tunis	6	4
Site 14	Tunisia, Sousse	0	0
Site 15	Bulgaria, Sofia	0	0
Site 16	Germany, Frankfurt	0	0

Supplementary Table 2. Coagulation parameters prothrombin time (PT), activated partial thromboplastin time (aPTT), D-dimer, thrombin-antithrombin-III-complex (TAT) and prothrombin fragment 1 and 2 (F1+2) throughout the study period. Results are expressed as median (IQR).

	Predose	Day 1	Day 2	Day 4	Day 7	Day 10	Day 14	Day 49
PT, sec	120 (120-120)	15.40 (14.50-15.90)	15.30 (14.4-16.7)	17.80 (16.5-25.8)	26.70 (21.8-60)	36.10 (30.3-120)	120 (120-120)	120 (120-120)
aPTT, sec	120 (120-120)	37 (33.3-37.5)	37 (32-37)	32 (32-34)	38 (35-120)	48 (41-120)	120 (93-120)	120 (120-120)
D-dimer, mg/L	0.220 (0.17-0.24)	0.220 (0.17-0.29)	0.26 (0.22-0.58)	0.243 (0.22-0.35)	0.22 (0.16-0.22)	0.22 (0.16-0.26)	0.22 (0.22-0.32)	0.22 (0.16-0.25)
TAT, µg/L	4.4 (3.1-15.5)	4.6 (3.5-7.55)	4.65 (3.2-14.3)	3.5 (2.7-8.7)	3.1 (2.7-4.05)	4.2 (3-10.4)	4.05 (3.45-8.85)	3.25 (2.80-4.4)
F1+2, pmol/L	230 (179-664)	275.5 (158.5-445.5)	173 (144-400)	202 (143-202)	226.5 (151.5-366.5)	347 (265.5-347)	241.5 (180.5-299.5)	218.5 (176-282)

Abbreviations: PT, prothrombin time; aPTT, activated partial thromboplastin time; TAT, thrombin-antithrombin-III-complex, F1+2, prothrombin fragment 1 and 2, and D-dimer

Thrombosis and Haemostasis | DOI 10.1055/a-2715-2994 | © The Author(s).
